# Special Issue: “Parasitic Infection and Host Immunity”: Editorial

**DOI:** 10.3390/microorganisms11041027

**Published:** 2023-04-14

**Authors:** Debora Decote-Ricardo, Danielle de Oliveira Nascimento, Leonardo Freire-de-Lima, Alexandre Morrot, Celio Geraldo Freire-de-Lima

**Affiliations:** 1 Instituto de Veterinária, Universidade Federal Rural do Rio de Janeiro, Rio de Janeiro 23890-000, Brazil; decotericardo@ufrrj.br (D.D.-R.); daniongabi@gmail.com (D.d.O.N.); 2 Instituto de Biofisica Carlos Chagas Filho, Universidade Federal do Rio de Janeiro, Rio de Janeiro 21941-170, Brazil; leolima@biof.ufrj.br; 3 Faculdade de Medicina, Universidade Federal do Rio de Janeiro, Rio de Janeiro 21044-020, Brazil; 4 Instituto Oswaldo Cruz, FIOCRUZ, Rio de Janeiro 21040-360, Brazil

Parasite–host interactions depend on a complex interplay between the metabolism of the parasite, their antigens, and the host immune response system [1,2]. In this Special Issue, we present various contributions on this topic, reporting extremely relevant issues in the interaction of different parasites with the host immunity.

One of these issues is the resistance to antimalarial treatments, which is already well documented for infection with *P. vivax* [3,4,5,6,7,8]. However, the molecular markers involved in the development of this resistance are not yet characterized [9]. The increase in pfmrps1 expression, a multidrug resistance-associated protein, is associated with antimalarial resistance. Polymorphisms in this gene are related to in vivo selection after treatment [10]. Interestingly, Yin et al. [4] show an association between pvmrp1 and the development of *P. vivax* resistance to antimalarial drugs. They found some unreported point mutations of pvmrp1, from collected samples, and S354N substitution which may lead to resistance to chloroquine in *P. vivax*. An analysis of the 29 polymorphisms of pvmrp1 within the 166 global isolates has revealed low nucleotide diversity and high haplotype diversity under purifying selection. However, analyzing the correlation between sensitivity/insensitivity to chloroquine and mutations has not showed any association between an increase in the number of copies of pvmep1 and its susceptibility to antimalarial drugs.

Relevant to the topic, Goo, Y. K. et al. [11] made a valuable contribution by discussing the biological and pathophysiological aspects of malaria caused by *P. vivax* from the perspective of the subtelomeric vir multigene superfamily. In *P. falciparum*, the var gene family encoding PfEMP1 protein plays a key role in antigenic variation and cytoadherence [12]. The similar *vir* multigene from *P. vivax* has been studied as an important superfamily gene that takes part in the pathogenesis of vivax malaria besides triggering the host immune activation [13]. Approximately 50% of the population infected by *P. vivax* have antibodies against the VIR protein, but the cellular response to this protein is not equally efficient. The number of studies on naturally acquired immune-response-induced VIR proteins in field populations from vivax malaria-endemic areas has been limited. Thus far, several studies have demonstrated the capacity of VIR proteins for mediating cytoadherence and immune response activation.

In addition, Martins et al. [14] have evaluated the role of SIRPα receptors in Plasmodium-induced immunosuppression. This receptor is recognized as mediating the inhibitory signals of innate immunity functions [15,16,17]. Thus, the group investigated the modulatory effects of Plasmodium crude extracts for SIRPα expression from innate immune cells. They incubated PBMC from healthy individuals in the presence of crude extracts obtained from both *P. falciparum* and *P. vivax*, using LPS as a control stimulus. The authors have observed that crude extracts derived from both parasites can induce increased SIRPα expression in monocytes, but not in dendritic cells. The authors pointed out that a study with malaria patients from the Brazilian Amazon region is currently in progress.

Another contribution in this field focused on important aspects of the disease caused by infection with freshwater parasitic worms from certain tropical and subtropical countries, such as schistosomiasis. One of the key characteristics of *Schistossoma mansoni* infection is the eosinophilia accompanied by the granuloma formation, resulting from the migration of inflammatory cells to the site of infection during the control of infection [18,19]. Malta et al. [20] reviewed important aspects of the immunopathology of *S. mansoni* infection from that perspective, taking into account relevant aspects such as the host’s immune response, the architecture of the granuloma, the recruitment and accumulation of eosinophils, the mechanisms of action of these cells, as well as their interaction with other cells using different animal models of infection. Although the primary functions of eosinophils in *S. mansoni* infection are still unclear, the authors stated that eosinophil cells play an important role in granuloma formation and the protection of parenchymal tissue, as they are critical for the destruction of entrapped eggs needed for the remodeling and repair of infected tissues.

In another contribution, Saïdi et al. [21] developed a direct immunofluorescence (DIF) technique that, instead of identifying Leishmania amastigotes, marks the presence of scattered antigens, as well as their degraded residues and soluble components, via the cytoplasm and plasma membrane of host cells. The need for new and more accurate methods for the diagnosis of cutaneous leishmaniasis is necessary since these gold methods have a detection margin of amastigotes between 60 and 80% [22,23]. The DIF assay applied to dermal scrapings was more sensitive than microscopy and as sensitive as PCR. Furthermore, the DIF assay using anti-*L. major* polyclonal IgG-FITC recognized other Leishmania species present in Tunisia.

In addition, Vargas-Villavicencio et al. [24] have reported that infections with *Toxoplasma gondii* yield a phenomenon where IgM, usually used to detect acute infection, ends up lasting for months. The authors also referred to chronic IgM, which makes the diagnosis difficult. Although this event has not yet been clarified, in this review, the authors propose various hypotheses based on original studies. In fact, there is evidence that gestational sexual hormones can prolong the acute infection. Thus, the reactivation of *T. gondii* could continuously induce low sustained IgM levels for long periods, as well as B-1 cell-derived natural antibodies and Ig class switch alterations caused by the parasite or cytokines taken from inflammatory host responses.

As presented in this Special Issue, these works highlight the complexity in this vast and intriguing field of research. Using different parasite infection models, in this Special Issue, the authors showed that host–pathogen interactions are specific for each pathogen, thus raising different challenges for the study of pathogenesis mechanisms, infection control, as well as the treatment and diagnostic methods of human parasitic diseases.
